# The Complex Charge Paradigm: A New Approach for Designing Electromagnetic Wavepackets

**DOI:** 10.1002/advs.201903377

**Published:** 2020-08-11

**Authors:** Liang Jie Wong, Demetrios N. Christodoulides, Ido Kaminer

**Affiliations:** ^1^ School of Electrical and Electronic Engineering Nanyang Technological University 50 Nanyang Ave Singapore 639798 Singapore; ^2^ CREOL The College of Optics & Photonics University of Central Florida Orlando FL 32816 USA; ^3^ Department of Electrical Engineering Technion Haifa 32000 Israel

**Keywords:** physical optics, singularities, nondiffracting waves, electrodynamics, optical wave shaping

## Abstract

Singularities in optics famously describe a broad range of intriguing phenomena, from vortices and caustics to field divergences near point charges. The diverging fields created by point charges are conventionally seen as a mathematical peculiarity that is neither needed nor related to the description of electromagnetic beams and pulses, and other effects in modern optics. This work disrupts this viewpoint by shifting point charges into the complex plane, and showing that their singularities then give rise to propagating, divergence‐free wavepackets. Specifically, point charges moving in complex space‐time trajectories are shown to map existing wavepackets to corresponding complex trajectories. Tailoring the complex trajectories in this “complex charge paradigm” leads to the discovery and design of new wavepacket families, as well as unprecedented electromagnetic phenomena, such as the combination of both nondiffracting behavior and abruptly‐varying behavior in a single wavepacket. As an example, the abruptly focusing X‐wave–a propagation‐invariant X‐wave‐like wavepacket with prechosen self‐disruptions that enhance its peak intensity by over 200 times–is presented. This work envisions a unified method that captures all existing wavepackets as corresponding complex trajectories, creating a new design tool in modern optics and paving the way to further discoveries of electromagnetic modes and waveshaping applications.

## Graphical abstract

This work presents a new paradigm in electromagnetic waveshaping—the complex charge paradigm—in which electromagnetic wavepackets can be engineered through the design of singularities moving along complex space‐time trajectories. This applies not only for existing wavepackets (such as the X‐wave), but also for novel electromagnetic effects with applications ranging from materials processing to radar in autonomous vehicles.

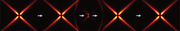

## 1 Introduction

Propagation‐invariant waves form a class of growing importance and have distinct electromagnetic profiles that remain unchanged with propagation. They range from nondiffracting modes, such as Bessel, Mathieu, Weber, Airy, and nonparaxial accelerating beams,^[^ ^1^, ^2^, ^3^, ^4^, ^5^, ^6^, ^7^, ^8^, ^9^, ^10^, ^11^, ^12^, ^13^ ^]^ to so‐called “space‐time” wavepackets^[^ ^14^, ^15^, ^16^, ^17^, ^18^, ^19^, ^20^, ^21^, ^22^, ^23^, ^24^, ^25^, ^26^, ^27^, ^28^, ^29^, ^30^ ^]^ whose propagation invariance arises from prespecified correlations between spatial and temporal frequency components of each mode in the wavepacket. Besides displaying a host of intriguing physical phenomena like self‐healing^[^ ^31^, ^32^, ^33^, ^34^ ^]^ and superluminal and subluminal travel,^[^ ^35^, ^36^, ^37^, ^38^, ^39^ ^]^ propagation‐invariant waves can potentially lead to greater speed and precision in scientific and industrial processes, such as particle manipulation,^[^ ^40^, ^41^, ^42^, ^43^, ^44^, ^45^ ^]^ laser micromachining,^[^ ^46^, ^47^, ^48^, ^49^ ^]^ communications,^[^ ^50^, ^51^ ^]^ and novel imaging techniques.^[^ ^52^, ^53^, ^54^ ^]^ Beyond these propagation‐invariant modes and the standard focused beams, there are still more classes of electromagnetic waves, including beams carrying topological charge,^[^ ^55^, ^56^, ^57^ ^]^ beams containing intricate structures like vortex and field‐line loops,^[^ ^58^, ^59^, ^60^, ^61^, ^62^, ^63^, ^64^, ^65^ ^]^ and autofocusing beams.^[^ ^66^ ^]^ This enormous diversity of electromagnetic modes raises the fundamental question as to whether there exists an overarching paradigm that unites them, and that can be leveraged to achieve greater versatility in controlling their dynamics.

Here, we take a step toward answering this crucial question by introducing the complex charge paradigm, in which a propagating electromagnetic wavepacket can be obtained as the result of a charge (or charge distribution) located in complex space‐time, and whose position is in general a complex analytic function of time. Unlike the complex source point method,^[^ ^25^, ^67^, ^68^, ^69^, ^70^, ^71^, ^72^, ^73^, ^74^, ^75^, ^76^, ^77^ ^]^ we deal not with stationary complex dipoles at one frequency, but instead a collection of arbitrarily moving complex charges, which we show can capture both nondiffracting as well as quasi‐nondiffracting wavepacket behavior using exact, fully closed‐form expressions. This allows us, for instance, to predict novel classes of wavepackets that combine both nondiffracting and abruptly focusing or self‐disrupting behavior. In contrast, the complex source point method uses a single‐frequency dipole source fixed in space and time to model a specific focused electromagnetic beam (it also cannot capture propagation‐invariant waves). Altogether, our new method could help capture the rich variety of optical wavepackets under a single unified theory, and alter the way we design specially‐shaped wavepackets.

We also show that the new closed‐form wavepacket expressions are readily used to design the experimental scheme needed for generating such wavepackets. Specifically, the expressions automatically yield the exact physical current distributions needed to generate the desired wavepackets. The needed current distributions can then be achieved through means such as appropriately designed metasurfaces (e.g., for generation at terahertz frequencies), spatial light modulators, or tunable millimeter‐wave antenna arrays. Using nonparaxial electromagnetic simulations for a finite bandwidth source, we show that these pulses can be realized using existing electromagnetic sources. This makes the complex charge paradigm a powerful tool for discovering new electromagnetic modes with applications ranging from materials processing to radar in autonomous vehicles.

## 2 Results

The Liénard–Wiechert potentials are known to provide solutions to the electromagnetic field of an arbitrarily moving point charge.^[^ ^78^ ^]^ As we will show, the electromagnetic fields resulting from a charge moving in complex space‐time, as opposed to real space‐time, are exact, physical solutions to the source‐free Maxwell's equations (provided that they contain no singularities). This ability to discover and construct electromagnetic wavepackets by simply defining the trajectory of a complex point charge—as opposed to more traditional methods, such as using a summation of plane waves—adds a novel, powerful tool to the arsenal of electromagnetic waveshaping theory.

As an example, we present a class of electromagnetic pulses in 3+1D—the abruptly focusing X‐wave—that exhibits both propagation‐invariant behavior and strong‐focusing behavior. For most of its propagation, the abruptly focusing X‐wave maintains a practically propagation‐invariant field profile, almost identical to the regular, nondiffracting X‐wave. At specific points in space‐time, however, the abruptly focusing X‐wave strongly focuses to an intense, highly localized hotspot, at which its peak intensity is enhanced by more than 200 times (and even more with appropriate parameter choices). It is important to note that the abruptly focusing X‐wave can exist in free space—or more generally, in a uniform, linear material medium—and its localized focusing behavior is not a result of material inhomogeneities or nonlinearities. By considering more general complex charge trajectories, we predict the existence of other novel types of self‐disrupting nondiffracting beams, which can display both symmetric and asymmetric behavior about one or multiple points of self‐disruption.

From Maxwell's equations, we obtain the electromagnetic fields of a moving point charge—whether in real^[^ ^78^ ^]^ or complex space‐time—as

(1) $$E=Re\left\{\frac{E_{0}}{R^{2}\eta^{3}}L\right\},\;B=\frac{1}{c}Re\left\{n\times E\right\}$$

where ***R*** = ***r***(*t*) − ***r***_0_(*t*′) ≡ (*R_x_*,*R_y_*,*R_z_*),***n*** ≡ ***R***/*R*, $R\;=\sqrt{R_{x}^{2}+R_{y}^{2}+R_{z}^{2}}\;,$ *η* = 1 − ***n*** · ***v***_0_(*t*′)/*c*, *c* being the speed of light in the uniform, linear medium in question. ***r***(*t*) is the location of the observer at time *t*, ***r***_0_(*t*′) = (*x*_0_(*t*′),*y*_0_(*t*′),*z*_0_(*t*′)) is the location of the charged particle at retarded time *t*′, which satisfies the equation *c* ^2^(*t* − *t*′)^2^ = *R* ^2^. The particle is assumed to travel at velocity ***v***_0_(*t*′) ≡ *d**r***_0_(*t*′)/*dt*′ and acceleration ***a***_0_(*t*′) ≡ *d**v***_0_(*t*′)/*dt*′. In Equation (1), ***L*** ≡ ***n*** × [(***n*** − ****β****) × ****α****] + (***n*** − ****β****)(1 − *β* ^2^), where ****β**** ≡ ***v***_0_(*t*′)/*c*, ****α**** ≡ *R**a***_0_(*t*′)/*c* ^2^, and *E*_0_ is an arbitrary constant whose value is simply *q*/4*πε*_0_—where *q* is the charge and *ε*_0_ the material permittivity—if Equation (1) is intended to simply describe the electromagnetic field of an actual charged particle moving in real space‐time (in which case all variables would have purely real values). In the Lorenz gauge, we find that the vector and scalar potentials corresponding to Equation (1) are given by the Liénard–Wiechert potentials

(2) $$\psi =\frac{E_{0}}{\eta R},\;A=\frac{E_{0}}{\eta R}\frac{\beta }{c}$$

**Figure 1:**
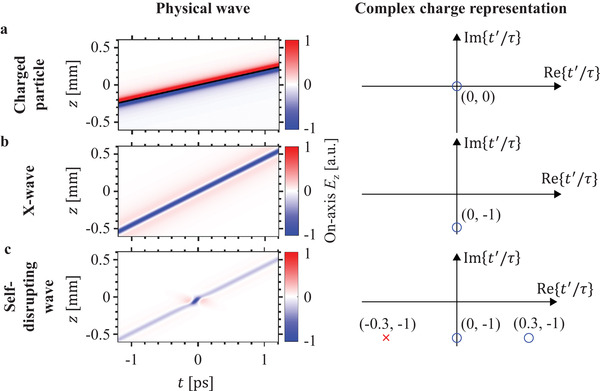
The complex charge paradigm. New and existing electromagnetic wavepackets can be predicted by specifying the trajectory *r*_0_(*t*′) of a charged particle in complex space‐time. The left panels show the *z*‐directed on‐axis electric field of various electromagnetic waves in real space‐time, whereas the right panels show the corresponding zeros (blue circles) and poles (red crosses) of the complex charge trajectory function, where we have used *τ* ≡ 0.1251 ps as a scaling factor. Panel (a) corresponds to the case of a physical charge traveling at *v* = 0.67*c* (trajectory indicated by black line). Panel b) corresponds to the case of a complex charge traveling at *v* = 1.5*c*, yielding the nondiffracting X‐wave. Panel (c) is the same as (b) except that an extra pole and zero have been included in the particle trajectory, yielding a new electromagnetic mode that is essentially nondiffracting except for a self‐disruption around *t* = 0 in the form of a kink of enhanced field intensity. The electromagnetic modes here correspond to the standard Liénard–Wiechert formulation in Equation (1).

If all these functions are real‐valued, (Equations (1) and (2) yield the fields and potentials of a moving point charge. To find the most general family of electromagnetic wavepackets, we allow the retarded time *t*′ to take complex values and ***r***_0_(*t*′), ***v***_0_(*t*′), ***a***_0_(*t*′) to be complex analytic functions, resulting in ***R***, *R*, ***n***, *η*, etc., all being complex. The ability to generate different electromagnetic fields by specifying different complex charge trajectories is illustrated in **Figure** **1**.

In the limiting case where the complex charge moves at a constant velocity, one obtains an electromagnetic field having the form of the well‐known nondiffracting X‐wave,^[^ ^14^, ^79^ ^]^ so‐named for its distinctive X‐shaped intensity profile. To show this, let us assume that the particle trajectory is given by $r_{0}\left(t\right)=\hat{z}bc\left(t-it_{0}\right)$, where *b* and *t*_0_ are real numbers, and $\hat{z}$ is the unit vector in the z‐direction. Note that this particle is located in complex space, and the retarded time *t*′ is in general also complex. Substituting this trajectory into Equation (2), we find after some algebra (see the Supporting Information Section 1 for details) that *ψ* and *A_z_* reduce to

(3) $$\psi_{X}=A_{Xz}\frac{c}{b}=\frac{\pm E_{0}}{\sqrt{\left(1-b^{2}\right)r^{2}+{\left(z-bct+icbt_{0}\right)}^{2}}}$$

where the “± ” sign arises from the multiple branches in the solution to the complex retarded time *t*′, and transverse components *A_Xx_* = *A_Xy_* = 0. Under the condition that |*b*| > 1, we indeed see as in ref. ^[^ ^14^ ^]^ that the rightmost side of Equation (3) has exactly the form of the 0th order nondiffracting X‐wave, with *b* playing the role of a normalized superluminal intensity peak velocity associated with this X‐wave. The case where *b* = 1.5 for the X‐wave is illustrated in Figure 1b.

Before proceeding further, we show that there is a complementary method in formulating the electromagnetic fields corresponding to a complex charge trajectory, by having the retarded time *t*′ of our complex charge fulfil the condition (i*z* + *ct*′)^2^ = (*x* − *x*_0_(*t*′))^2^ + (*y* − *y*_0_(*t*′))^2^ + (i*ct* − *z*_0_(*t*′))^2^, instead of the usual condition (*ct* − *ct*′)^2^ = (*x* − *x*_0_(*t*′))^2^ + (*y* − *y*_0_(*t*′))^2^ + (*z* − *z*_0_(*t*′))^2^. This technique, which can be loosely understood as a swap between time and space coordinates (specifically, i*ct* and *z*), has been used to predict new electromagnetic modes such as time‐diffracting wavepackets.^[^ ^26^, ^80^ ^]^ By applying this technique to our problem, we discover the following electromagnetic fields that also satisfy Maxwell's equations, and are thus valid electromagnetic solutions to the complex charge under consideration:

(4) $$\begin{array}{c} {\tilde{E}}_{⊥}=Re\left\{\frac{iE_{0}}{{\tilde{R}}^{2}{\tilde{\eta }}^{3}}\hat{z}\times \left(\tilde{n}\times \tilde{L}\right)\right\},\;\;{\tilde{E}}_{z}=Re\left\{\frac{E_{0}}{{\tilde{R}}^{2}{\tilde{\eta }}^{3}}\hat{z}\cdot \tilde{L}\right\}\\ {\tilde{B}}_{⊥}=\frac{1}{c}Re\left\{\frac{-iE_{0}}{{\tilde{R}}^{2}{\tilde{\eta }}^{3}}\hat{z}\times \tilde{L}\right\},\;\;{\tilde{B}}_{z}=Re\left\{\frac{E_{0}}{{\tilde{R}}^{2}{\tilde{\eta }}^{3}}\hat{z}\cdot \left(\tilde{n}\times \tilde{L}\right)\right\} \end{array}$$

with $\tilde{R}\equiv \left(x-x_{0}\left(t^{\prime }\right),\;y-y_{0}\left(t^{\prime }\right),\;ict-z_{0}\left(t^{\prime }\right)\right)$, $\tilde{n}\equiv \tilde{R}/\tilde{R}$, $\tilde{R}=\sqrt{{\tilde{R}}_{x}^{2}+{\tilde{R}}_{y}^{2}+{\tilde{R}}_{z}^{2}}\;,$ $\tilde{\eta }=\;1+\tilde{n}\cdot v_{0}\left(t^{\prime }\right)/c$, and $\tilde{L}\equiv \tilde{n}\times \left[\tilde{\alpha }\times \left(\tilde{n}+\beta \right)\right]-\left(\tilde{n}+\beta \right)\left(1-\beta^{2}\right)$, where ****β**** ≡ ***v***_0_(*t*′)/*c* and $\tilde{\alpha }\equiv \tilde{R}a_{0}\left(t^{\prime }\right)/c^{2}$. Here, the complex retarded time is obtained by solving the equation ${\left(iz+ct^{\prime }\right)}^{2}={\tilde{R}}^{2}\;\;$. *E*_0_ is an arbitrary constant. We obtain the vector potentials corresponding to Equation (4) as

(5) $$\psi =\frac{-iE_{0}}{\tilde{\eta }\tilde{R}}\beta_{z},\;A_{⊥}=\frac{E_{0}}{\tilde{\eta }\tilde{R}}\frac{\beta_{⊥}}{c},\;A_{z}=\frac{iE_{0}}{\tilde{\eta }\tilde{R}}\frac{1}{c}$$

As before, a charge moving at a constant velocity in complex space‐time yields X‐waves, but now under the condition that |*b*_0_| < 1 in the equation $r_{0}\left(t\right)=\hat{z}b_{0}c\left(t-t_{0}/b_{0}\right)$ (see the Supporting Information Section 1 for details). More generally, one can add an arbitrary number of poles and zeros (including in the other Cartesian directions $\hat{x}$ and $\hat{y}$) to the equation for ***r***_0_(*t*) to generate infinitely many new families of electromagnetic wavepackets. To distinguish this new formulation from the standard Liénard–Wiechert equations given by Equations (1) and (2), we will term Equations (4) and (5) as the time‐diffracting Liénard–Wiechert formulation. In the remainder of this paper, we will restrict our study to analytic trajectories of the form (complex analytic functions can generally be expressed this way)

(6) $$r_{0}\left(t\right)=\hat{z}b_{0}c\frac{\prod_{i}\left(t-t_{zi}\right)}{\prod_{j}\left(t-t_{pj}\right)}$$

where the zeros *t_zi_* and poles *t_pj_* have complex values, using the time‐diffracting Liénard–Wiechert formulation given by Equation (4).

**Figure 2:**
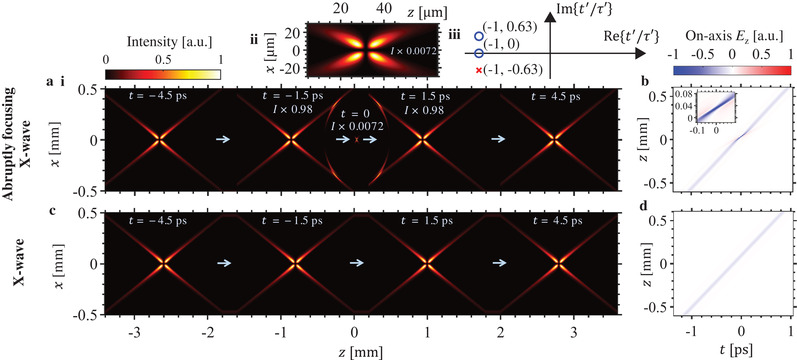
Abruptly focusing vortex X‐wave. The intensity profile (magnitude of Poynting vector in z‐direction) of a radially‐polarized abruptly focusing X‐wave is shown in (a), with (a(ii)) showing a magnified view of the high‐intensity region. This wavepacket corresponds to a complex charge trajectory (Equation (6)) with *b*_0_ = − 0.5 and zeros (blue circles) at *t*′ = −*τ*′ and *t*′ = (−1 + i0.63)*τ*′, and a pole (red cross) at *t*′ = (−1 −i0.63)*τ*′, as marked in (a(iii)), where the scaling factor *τ*′ ≡ 0.16678 ps. Panel b) shows the on‐axis longitudinal electric field *E_z_*. Panels c,d) show the profile of a regular nondiffracting X‐wave whose complex charge trajectory contains just one zero at *t*′ = −*τ*′. Note that both abruptly focusing and regular nondiffracting X‐waves are radially‐polarized here, so their *z*‐directed Poynting vector profiles form vortices with vanishing on‐axis (*x* = *y* = 0) intensity. Far from the focal plane (the focal plane being located at *z* = 32.4 µm), the abruptly focusing X‐wave behaves like a nondiffracting wave. Near the focal plane, the profile of the abruptly focusing X‐wave changes rapidly, achieving intensity enhancements of over 200 times at the focus. The electromagnetic modes here correspond to the time‐diffracting Liénard–Wiechert formulation in Equations (4) and (5).

The abruptly focusing X‐wave shown in **Figure** **2**a corresponds to a complex charge trajectory with two zeros and one pole in Equation (6), as marked on the complex plane in Figure 2a(iii). In contrast, the regular nondiffracting X‐wave in Figure 2c corresponds to a complex charge trajectory containing only one zero. Whereas the nondiffracting X‐wave remains propagation invariant throughout space‐time, the abruptly focusing X‐wave is quasi‐nondiffracting in the sense that its field profile closely resembles its nondiffracting counterpart for most of the propagation, but is also capable of abruptly focusing and abruptly defocusing before resuming a practically invariant profile for the rest of its propagation. As seen in Figure 2a(ii), a hotspot with an intensity enhancement of over 200 times can be thus achieved. Comparing Figure 2b,d, we see that the longitudinal electric fields of abruptly focusing and regular nondiffracting X‐waves are practically identical except for the tightly focused kink in the field of the former. The abruptly focusing X‐wave thus combines the nondiffracting attributes of propagation‐invariant waves with the high intensity hotspots of tightly focused beams.

**Figure 3:**
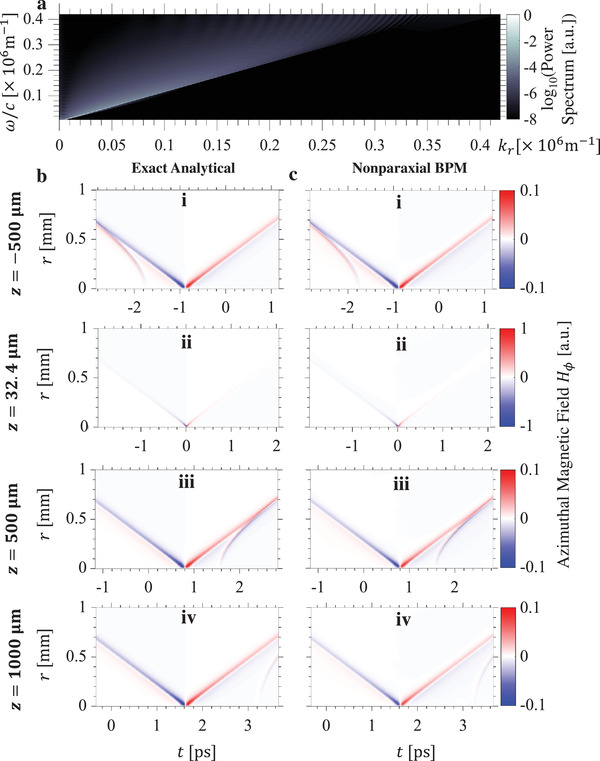
Power spectrum and propagation of finite‐aperture, band‐limited abruptly focusing X‐waves. a) Power spectrum of the abruptly focusing X‐wave shown in Figure 2 at *z* = −500 µm (*z* = 32.4 µm being the focal plane). In (b,c), we see excellent agreement between exact analytical expressions of the abruptly focusing X‐wave, and the predictions of a numerical nonparaxial beam propagation method (BPM) based on the initial beam profile shown in (c(i)). Parameters are exactly the same as in Figure 2a. In the nonparaxial BPM scenario, shown in (c), an additional transverse Gaussian envelope of waist radius 5 mm was superposed on the profile to simulate the effects of a finite aperture. Additionally, an artificial bandpass filter admitting only frequencies between 0.3 and 20 THz was imposed to show that the abruptly focusing X‐wave can be accurately produced with a finite bandwidth source.

In **Figure** **3**, we show that it is possible to accurately generate an abruptly focusing X‐wave using a source that is limited in both physical size as well as frequency content. Specifically, we use a nonparaxial beam propagation method, which numerically solves the exact Maxwell's equations, to propagate the initial pulse whose frequency content is shown in Figure 3a, and whose spatiotemporal profile (for the azimuthal magnetic field component *H*_*ϕ*_) is shown in Figure 3c(i). Figure 3b(i–iv) shows the spatiotemporal evolution of the wavepacket assuming an infinite bandwidth and transverse extent. The results of Figure 3c(i–iv) show the spatiotemporal evolution of the same wavepacket when modulated by a transverse Gaussian envelope of waist radius 5 mm, and restricted to carry frequencies between 0.3 and 20 THz with a spectral resolution of 23.1 GHz, a spectral range and resolution experimentally achievable with existing technology.^[^ ^81^, ^82^, ^83^, ^84^, ^85^, ^86^ ^]^ The agreement between the results of Figure 3b,c indicates the viability of realizing the abruptly focusing X‐wave with sources of finite sizes and bandwidths. As we show in the Supporting Information Section 2, it is possible to realize the abruptly‐focusing X‐wave with even narrower‐band sources.

**Figure 4:**
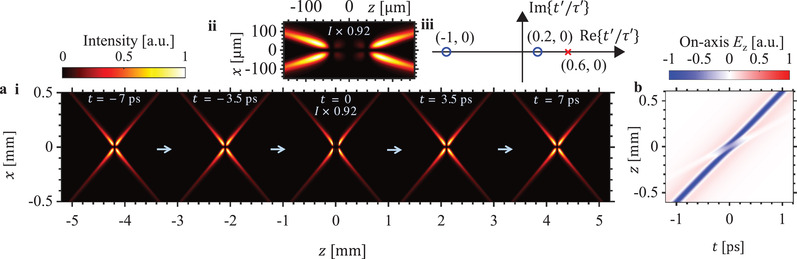
Symmetric self‐disrupting X‐wave. The intensity profile (magnitude of Poynting vector in *z*‐direction) of a radially‐polarized self‐disrupting X‐wave is shown in (a). This wavepacket corresponds to a complex charge trajectory (Equation (6)) with *b*_0_ = −0.5 and zeros (blue circles) at *t*′ = −*τ*′ and *t*′ = 0.2*τ*′, and a pole (red cross) at *t*′ = 0.6*τ*′, as marked in (a(iii)), where the scaling factor *τ*′ ≡ 0.16678 ps. The abrupt disruption in the intensity profile in (a(i)) (zoom‐in of *t* = 0 in (a(ii))) is also reflected in the behavior of on‐axis longitudinal electric field *E_z_* in (b). It is noteworthy that the behavior of the quasi‐nondiffracting wavepacket is symmetric about the disruption. The electromagnetic modes here correspond to the time‐diffracting Liénard–Wiechert formulation in Equations (4) and (5).

**Figure 5:**
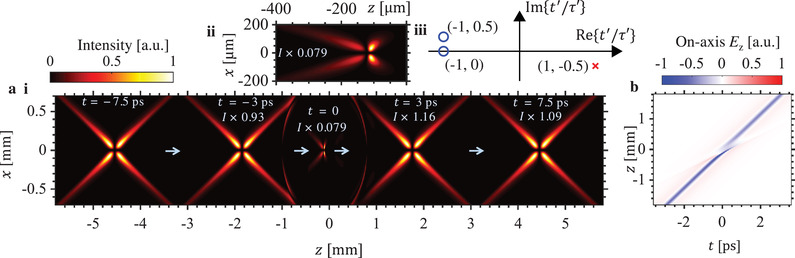
Asymmetric self‐disrupting X‐wave. The intensity profile (magnitude of Poynting vector in *z*‐direction) of a radially‐polarized self‐disrupting X‐wave is shown in (a). This wavepacket corresponds to a complex charge trajectory (Equation (6)) with *b*_0_ = −0.5 and zeros (blue circles) at *t*′ = − *τ*′ and *t*′ = (−1 + i0.5)*τ*′, and a pole (red cross) at *t*′ = (1 − i0.5)*τ*′, as marked in (a(iii)), where the scaling factor *τ*′ ≡ 0.16678 ps. The abrupt disruption in the intensity profile in (a(i)) (zoom‐in of *t* = 0 in (a(ii)) is also reflected in the behavior of on‐axis longitudinal electric field *E_z_* in (b). It is noteworthy that the behavior of the quasi‐nondiffracting wavepacket is asymmetric about the disruption, in contrast to the symmetric behavior in Figure 4. The electromagnetic modes here correspond to the time‐diffracting Liénard–Wiechert formulation in Equations (4) and (5).

The complex particle trajectory defined by Equation (6) can be adjusted not only to control the location and time of the abrupt focusing behavior, but also to induce other kinds of localized disruptions besides abrupt focusing. In **Figure** **4**, we see that by appropriately choosing the zeros and poles in Equation (6), a self‐disrupting behavior can be induced. The on‐axis longitudinal field *E_z_*, which is critical in applications such as laser‐driven particle acceleration^[^ ^87^, ^88^, ^89^ ^]^ and laser‐induced electron pulse compression,^[^ ^90^, ^91^ ^]^ weakens momentarily before strengthening again and resuming a practically propagation invariant behavior. **Figure** **5** shows that adjusting the complex charge trajectory can also affect the behavior of the wavepacket as it recovers from the self‐disruption, by showing a combination of zeros and poles that changes the symmetric behavior in Figure 4 to an asymmetric evolution in space‐time.

## 3 Discussion

A viable option for experimentally realizing the wavepackets presented here at optical frequencies could be the use of pre‐engineered phase masks and amplitude masks or spatial light modulators, in either real space or Fourier space.^[^ ^92^, ^93^ ^]^ The ability to design wavepackets by programming specific values at specific frequency‐wavevector combinations has already been demonstrated in setups that combine elements from spatial beam modulation and ultrafast pulse shaping.^[^ ^22^, ^23^ ^]^ Another possible realization would be through time‐dependent current distributions. Specifically, one can compute the current distribution *Re*{***J**_s_*} in the *x*‐*y* plane at a given *z* via the expression ***J**_s_* = *z* × ***B***/*μ*_0_, where *μ*_0_ is the material permittivity. The radiation from such a current distribution will generate the electromagnetic wavepacket whose magnetic field is described by ***B***. An ordinary pulsed laser incident on a metasurface or nanoantenna array could then be used to induce the desired current density modulations. These wavepackets can also be realized at millimeter‐wave or microwave frequencies, through the use of metasurfaces or configurable antenna arrays.^[^ ^94^, ^95^, ^96^, ^97^ ^]^ In particular, one can imagine an array of patch antennas whose output digitally specifies the required current distribution *Re*{***J**_s_*} (e.g., through digital‐to‐analog converters), allowing simultaneous spatial and temporal shaping of the output wavefront to its desired form. The range of alternative approaches continues to be broadened by the discovery of new spatiotemporal pulse shaping methods.^[^ ^98^, ^99^ ^]^

The complex charge paradigm has significant qualitative advantages compared to existing methods of wavepacket design. The complex charge paradigm is to date the only method capable of designing wavepackets that exhibit both nondiffracting behavior and rapidly‐varying behavior, a phenomenon that has never been predicted or observed before. The complex charge paradigm has significant quantitative advantages as well: it is able to describe the entire waveform by a small set of complex parameters (describing the complex trajectory), instead of a massive array of field amplitudes and phases, as is required by conventional methods like plane wave decomposition.

The examples presented in Figures 2, 3, 4, 5 show that many different types of locally disruptive behavior can be synthesized even when we restrict ourselves to the case of 2 zeros and 1 pole for a complex charge trajectory described by Equation (6). These novel examples, which have so far eluded discovery using all known waveshaping techniques, testify to the power and robustness of the complex charge paradigm in predicting new electromagnetic modes. Once discovered using the complex charge paradigm, these novel modes can also be reverse engineered using other methods, such as plane wave decomposition, for the purpose of experimental realization using conventional equipment like spatial light modulators and phase masks. It is possible to introduce additional local disruptions or design local disruptions of more complex natures by introducing even more poles and zeros in Equation (6), by adding poles and zeros in other Cartesian directions, or by working with analytic (in the Cauchy–Riemann sense) trajectories of other forms. For example, we can design electromagnetic beams containing multiple self‐disrupting (e.g., abruptly focusing and defocusing) points at user‐specified space‐time locations, by designing the complex charge trajectory that contains the appropriate pole‐zero pairs. Instead of considering a single complex charge, it is also possible to extend the theory to arbitrary, finite‐sized charge distributions.

The complex charge paradigm opens many questions. For instance, what fundamental rules apply here and what sets of poles and zeros are “allowed” in order to produce electromagnetic fields free of singularities in real space‐time? What types of complex charge trajectories must be chosen to generate and manipulate other well‐known beams, such as the Airy beam? Do the laws of electromagnetism admit a unifying paradigm, where all singular points in an electromagnetic field can be understood to arise from the presence of sources in real space‐time, while all nonsingular parts of the electromagnetic field can be interpreted as arising from the presence of sources that lie out of the real space‐time plane, in complex space‐time?

## 4 Conclusion

In conclusion, we have presented the complex charge paradigm: the use of a charge (or charge distribution) that exists in complex space‐time and whose trajectory can be controlled at will to generate both existing and new families of electromagnetic wavepackets. As an example, we have shown that the choice of a complex charge moving at a constant velocity corresponds to the well‐known nondiffracting X‐wave. We have also shown that different types of localized disruptions, including strong‐focusing and self‐disruptive behavior, can be introduced and controlled by altering the trajectory of the complex charged particle. We presented three examples of novel electromagnetic wavepackets that exhibit both propagation‐invariant and abruptly changing characteristics: 1) the abruptly focusing X‐wave, which focuses down to intensity hotspots with intensity enhancements of over 200 times; 2) the symmetric self‐disrupting X‐wave, whose longitudinal on‐axis electric field momentarily weakens and then recovers its original profile in a way that is symmetric about the disruption; and 3) the asymmetric self‐disrupting X‐wave, whose longitudinal electric field changes in a asymmetric way about the localized disruption. Using nonparaxial electromagnetic simulations for a finite bandwidth source, we further showed that these pulses can be realized using existing electromagnetic sources. These wavepackets are unique in exhibiting both propagation‐invariant and abruptly changing behavior, which are typically thought to be mutually exclusive. The complex charge paradigm introduces a unifying paradigm for electromagnetic waves, where we can capture existing modes of light and predict new ones by shifting charge singularities from real to complex space‐time. These charges can be embedded in inhomogenous and even nonlinear media, providing a robust way to predict new phenomena in all kinds of platforms.

## Conflict of Interest

The authors declare no conflict of interest.

## Supporting information

Supporting Information
