# Supplementary material for: The Complex Charge Paradigm: A New Approach for Designing Electromagnetic Wavepackets
Source: Adv Sci (Weinh). 2020 Aug 11;7(19):1903377. doi: 10.1002/advs.201903377 (PMC7539223; doi:10.1002/advs.201903377)
Supplement: Supplementary file 1 — Supporting Information [file ADVS-7-1903377-s001.pdf]

Copyright WILEY-VCH Verlag GmbH & Co. KGaA, 69469 Weinheim, Germany, 2020.

ADVANCED SCIENCE
Open Access

Supporting Information

for *Adv. Sci.*, DOI: 10.1002/advs. 201903377

The complex charge paradigm: A new approach for designing electromagnetic wavepackets

Liang Jie Wong, Demetrios N. Christodoulides, and Ido Kaminer*

Supporting Information

The complex charge paradigm: A new approach for designing electromagnetic wavepackets

Liang Jie Wong, Demetrios N. Christodoulides, and Ido Kaminer*

1. Nondiffracting X-waves from the Liénard-Wiechert formulations

The purpose of this section is to present a derivation for the electromagnetic potential corresponding to the nondiffracting X-wave (namely, Equation (3) of the main text) from the standard formulation of the Liénard-Wiechert potentials, given by Equations (1) and (2) of the main text; and from the time-diffracting formulation of the Liénard-Wiechert potentials, given by Equations (4) and (5) of the main text. We see that in both standard and time-diffracting formulations, the complex charge that yields a nondiffracting X-wave is one that moves at constant velocity. However, whereas the charge has a superluminal velocity in the standard case, the charge has a subluminal velocity instead in the time-diffracting case. Unless otherwise stated, all notation used here is as defined in the main text.

1.1 Nondiffracting X-waves from the standard Liénard-Wiechert formulation

Consider a complex charge that executes the trajectory

$$\boldsymbol{r}_0(t') = \hat{z}bc(t' - \mathrm{i}t_0) \equiv \hat{z}z_0(t') \qquad (S1)$$

as a function of time t'. In the standard formulation, the retarded time t' and observation time t are related by the expression

$$(ct - ct')^2 = x^2 + y^2 + \left(z - z_0(t')\right)^2. \qquad (S2)$$

Solving for t' yields

$$t' = \frac{1}{1 - b^2}\left[t - \frac{b}{c} - b^2 it_0 \pm \sqrt{(1 - b^2)\frac{r^2}{c^2} + \left(-bt + \frac{z}{c} + bit_0\right)^2}\right]. \qquad (S3)$$

Substituting Equation (S1) into Equation (2) of the main text gives

$$\psi = A_z \frac{c}{b} = \frac{E_0}{(ct - ct') - b\big(z - z_0(t')\big)}, \qquad \boldsymbol{A}_\perp = 0. \tag{S4}$$

Substituting Equation (S3) into Equation (S4) leads us directly to Equation (3) of the main text. It should be noted that valid solutions require that the real part of t_0 is nonzero and that $|b| > 1$, corresponding to a superluminal charged particle in Equation (S1).

1.2 Nondiffracting X-waves from the temporally-diffracting Liénard-Wiechert formulation

Consider a complex charge that executes the trajectory

$$\boldsymbol{r}_0(t') = \hat{z} b_0 c(t' - t_0/b_0) \equiv \hat{z} z_0(t') \tag{S5}$$

as a function of time t'. In the temporally-diffracting formulation, the retarded time t' and observation time t are related by the expression

$$(\mathrm{i}z + ct')^2 = x^2 + y^2 + \big(ict - z_0(t')\big)^2. \tag{S6}$$

Solving for t' yields

$$t' = \frac{1}{1 - b_0^2}\left[-i\frac{z}{c} - ib_0 t - b_0 t_0 \pm \sqrt{(1 - b_0^2)\frac{r^2}{c^2} + \left(ib_0\frac{z}{c} + it + +t_0\right)^2}\,\right]. \tag{S7}$$

Substituting Equation (S5) into Equation (5) of the main text gives

$$\psi = -A_z b_0 c = \frac{-iE_0 b_0}{(iz + ct') + b_0\big(ict - z_0(t')\big)}, \qquad \boldsymbol{A}_\perp = 0. \tag{S8}$$

Substituting Equation (S7) into Equation (S8) gives

$$\psi = \frac{E_0}{\pm\sqrt{(1 - b_0^{-2})r^2 + (z + ct/b_0 - ict_0/b_0)^2}}, \tag{S9}$$

which is exactly equivalent to the rightmost side of Equation (3) of the main text when we set $b = -1/b_0$. It should be noted that valid solutions require that the real part of t_0 is nonzero and that $|b_0| < 1$, corresponding to a subluminal charged particle in Equation (S5).

2. Abruptly-focusing X-wave using a narrower terahertz band (0.3-10 THz)

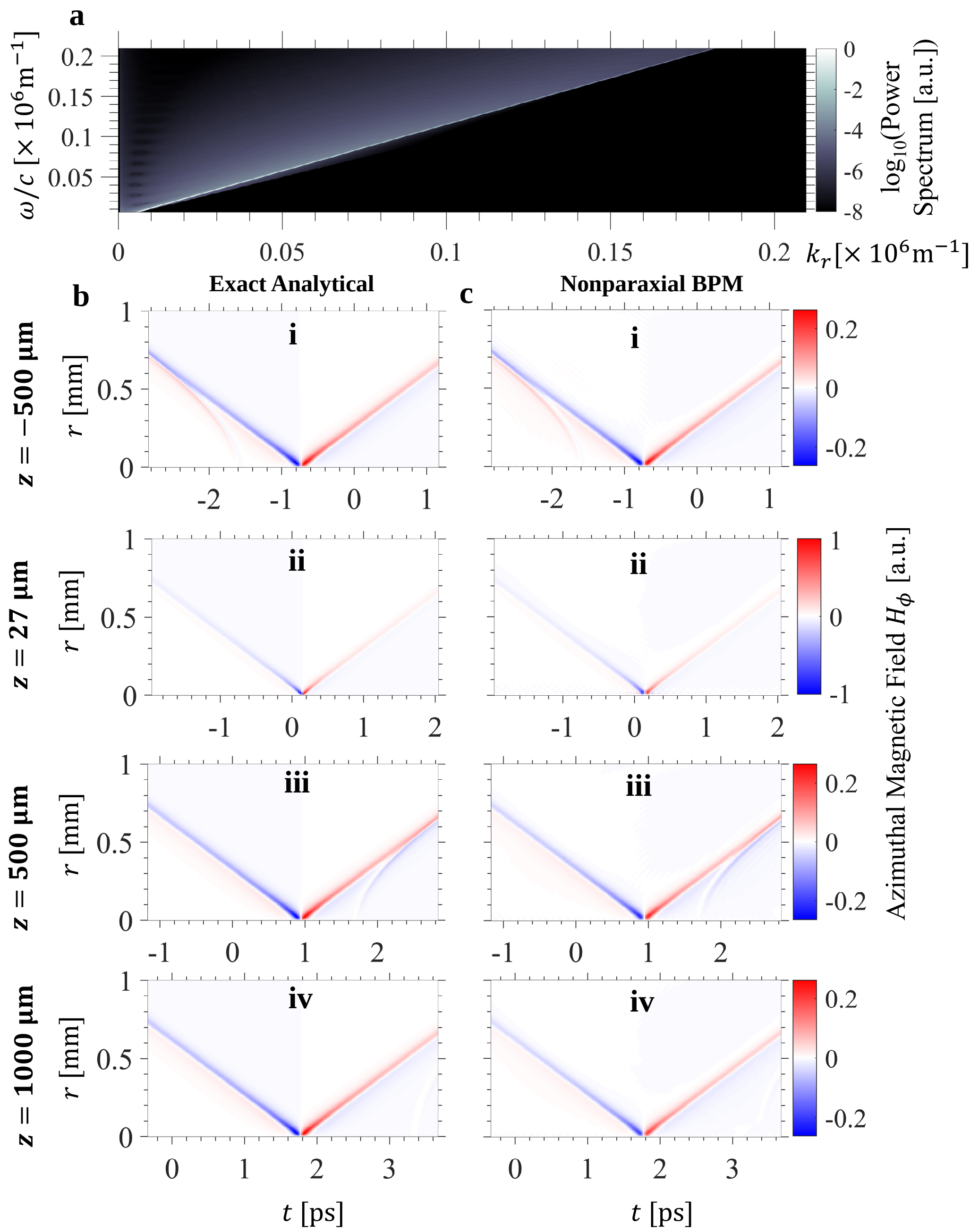

Figure S1. Power spectrum and propagation of an abruptly focusing X-wave realized using a narrower terahertz bandwidth (0.3-10 THz) . (a) Power spectrum of the abruptly focusing X-wave at z = -500 µm (z = 27.0 µm being the focal plane). In **b** and **c**, we see excellent agreement between exact analytical expressions of the abruptly focusing X-wave, and the predictions of a band-limited numerical nonparaxial beam propagation method (BPM) based on the initial beam profile shown in **c(i)**. Parameters are exactly the same as in Figure 2a, except the zeros now occur at $t' = -\tau'$ and $t' = (-1 + \mathrm{i}0.55)\tau'$, and the pole at $t' = (-1 - \mathrm{i}0.55)\tau'$, where the scaling factor $\tau' \equiv 0.16678$ ps. In the nonparaxial BPM, whose results are shown in **c**, an additional transverse Gaussian envelope of waist radius 5 mm was superposed on the profile to simulate the effects of a finite aperture. Additionally, an artificial bandpass filter admitting only frequencies between 0.3 THz and 10 THz was imposed to show that the abruptly focusing X-wave can be accurately produced with a source that has an even narrower (compared to Figure 2 of the main text) finite bandwidth.

The objective of this section is to clarify that the phenomena predicted via the complex charge paradigm are not restricted to broadband spectra. In fact, the complex trajectory can always be designed such that the resulting wavepacket can be realized using an emitter of limited bandwidth. Limiting the bandwidth of the resulting wavepacket in this way could lead to diminished properties – such as a weaker focus in the case of the abruptly focusing X-wave – but it is important to note that the complex charge paradigm remains a robust tool for discovery and design even when the desired bandwidth is limited.

In Fig. 3 of the main text, we saw that the abruptly focusing X-wave can be accurately reproduced even after imposing an artificial bandpass filter admitting only frequencies between 0.3 THz and 20 THz. This suggests that it is possible to experimentally realize the abruptly focusing X-wave using a broadband source that spans the frequency range 0.3-20 THz. Such a broadband source has been demonstrated before [84].

It is important to note that the abruptly focusing X-wave can, in fact, be demonstrated using sources with a narrower bandwidth. Adjusting the trajectory of the complex charge through the zeros and pole of its trajectory function, we find that we can realize an abruptly focusing X-wave from a source limited to the frequency range 0.3-10 THz, which we show in Fig. S1. Because of the reduced bandwidth, the intensity enhancement at the focus is about 20, but this is still considerable. With careful design – in terms of both the charge trajectory as well as the spectral resolution – it is possible to reduce the required bandwidth even further. The complex charge paradigm combines the best of two aspects: On the one hand, the novel broadband phenomena it predicts motivates applications for, and further research into, the development of broadband/super-continuum light sources. On the other hand, the complex charge paradigm is not inherently linked to any range of frequencies, and can just as readily be used to design phenomena that can be realized with narrower-band sources.
